# Dynamic mortality prediction in critically Ill children during interhospital transports to PICUs using explainable AI

**DOI:** 10.1038/s41746-025-01465-w

**Published:** 2025-02-17

**Authors:** Zhiqiang Huo, John Booth, Thomas Monks, Philip Knight, Liam Watson, Mark Peters, Christina Pagel, Padmanabhan Ramnarayan, Kezhi Li

**Affiliations:** 1 Institute of Health Informatics, University College London, London, UK; 2 Department of Population Health Sciences, King’s College London, London, UK; 3 Digital Research Innovation and Virtual Environment (DRIVE), Great Ormond Street Hospital, London, UK; 4 University of Exeter Medical School, Exeter, UK; 5 Children’s Acute Transport Service (CATS), Great Ormond Street Hospital, London, UK; 6 UCL Great Ormond Street Institute of Child Health, University College London, London, UK; 7 Clinical Operational Research Unit, University College London, London, UK; 8 Department of Surgery and Cancer, Imperial College London, London, UK; 9 Present Address: Wolfson Institute of Population Health, Queen Mary University of London, London, UK

**Keywords:** Paediatrics, Risk factors, Outcomes research, Paediatric research

## Abstract

Critically ill children who require inter-hospital transfers to paediatric intensive care units are sicker than other admissions and have higher mortality rates. Current transport practice primarily relies on early clinical assessments within the initial hours of transport. Real-time mortality risk during transport is lacking due to the absence of data-driven assessment tools. Addressing this gap, our research introduces the PROMPT (Patient-centred Real-time Outcome monitoring and Mortality PredicTion), an explainable end-to-end machine learning pipeline to forecast 30-day mortality risks. The PROMPT integrates continuous time-series vital signs and medical records with episode-specific transport data to provide real-time mortality prediction. The results demonstrated that with PROMPT, both the random forest and logistic regression models achieved the best performance with AUROC 0.83 (95% CI: 0.79–0.86) and 0.81 (95% CI: 0.76–0.85), respectively. The proposed model has demonstrated proof-of-principle in predicting mortality risk in transported children and providing individual-level model interpretability during inter-hospital transports.

## Introduction

Studies have shown that centralising specialised paediatric critical care in fewer centres has clear benefits. This approach helps deliver high-quality care at a lower cost while improving patient health outcomes^1,2^. Following the establishment of regional Paediatric Intensive Care Units (PICUs) in the United Kingdom, specialised Paediatric Critical Care Transport teams (PCCTs) were also developed. In England and Wales, 29 PICUs offer critical care services to over 11 million children under the age of 18^3^. The majority of transfers from other hospitals to PICUs are stabilised and transferred by PCCTs^4^. Despite being staffed by specialised and experienced paediatric clinicians, PCCTs face significant challenges in transporting critically ill children from acute general hospitals to PICUs, due to clinical complexity, staff-resource limitations, and time constraints in a small and extreme environment^5,6^.

Ensuring the safe transport of critically ill children to tertiary PICU centres is difficult, even for PCCT professionals. They often face challenges because transport conditions can change quickly, requiring them to act fast in response to any emergencies^7^, such as the acute deterioration of a child’s condition in a moving ambulance. Critically ill children are particularly vulnerable to preventable adverse events during inter-hospital transports, with incidents affecting up to 22% of such transfers^8,9^. Transported children exhibit longer PICU stays and a mortality rate of approximately 8% when requiring advanced care in tertiary PICUs, a figure higher than the mortality rate of other admissions^3^. With the development of digital and Artificial Intelligence (AI) technology, smart algorithms can predict critically ill patients’ outcomes for earlier intervention^10–12^. However, existing solutions that have been offered are primarily suitable for static ICUs and not for mobile environments. The key difference in mobile environments is the dynamic and unpredictable nature of patients, which introduces unique clinical and logistical challenges, such as wide range of severities of illness, varying environmental factors, limited space, and the need for continuous monitoring and rapid response during transit^3,13,14^. Therefore, PCCTs call for innovative and digital solutions to effectively mitigate these specific challenges in mobile critical care settings^15^.

During transport tasks, the team usually monitors bedside vital sign displays to spot unusual readings, such as prolonged drops in blood pressure or oxygen levels. Although continuous monitoring aids in identifying subtle physiological changes, the lack of real-time explanations complicates the understanding of multi-variable risk factors, compromising their capability to make immediate decisions^16^. Furthermore, while mortality indices like the Paediatric Index of Mortality 3 (PIM3) are very useful to estimate mortality risk made during early stabilisation, they only provide a “snapshot” of a patient’s condition, failing to capture dynamic progression during transport^17–19^. The real-time accuracy of mortality scores may be affected by the severity and interventions that occur post-stabilisation in the intensive care setting^7^. Therefore, the literature demands the development of diverse data-driven and AI-empowered solutions to the assessment of illness severity within the intensive care setting^20–22^.

Recent studies demonstrated the potential of Machine Learning (ML) and deep learning in predicting clinical outcomes through clinical data analysis^23–28^. For example, Sundrani et al. utilised deep learning to develop a model for predicting patient mortality based on continuous physiological data from emergency departments^29^. Lee et al. proposed a ML model to forecast postoperative mortality for surgical risk assessment with multi-centre validation^30^, and Hilton et al. introduced an ML pipelines to predict outcomes, including length of hospital stay and mortality rates^31^. Aiming to early recognise sepsis, Boussina assessed the impact of a deep-learning model for the early prediction of sepsis on patient outcomes^32^. Although these models have shown promise, their reliance on Electronic Health Records (EHR) or intermittently collected vital sign data limits their applicability to the real-time decision-making support system in the transport environment. Another challenge is that these models are often difficult to interpret, which makes it harder to provide personalised prognosis predictions for patients from different groups^33–36^.

In this work, we introduce PROMPT (Patient-centred Real-time Outcome monitoring and Mortality PredicTion), an explainable ML pipeline designed to dynamically assess severity of illness and 30-day mortality prediction for critically ill children during inter-hospital transports to the PICUs. The contributions of the research are three-fold:

1. We develop an end-to-end ML-based pipeline for predicting the 30-day mortality risk of transported critically ill children by incorporating continuous vital signs, EHR, and transport episode data;
2. Using real-time patient and transport data, the pipeline provides dynamic risks throughout the transport, supporting interventions and treatments;
3. Predicted risks using the PROMPT can be explained at the individual level within a variable time window, detailing how variable features influence the model output.

In prior research^37^, we investigated the distribution and progression of continuous vital sign data during inter-hospital transports by applying Z-scores to standardise the vital signs of children across different age groups. In this work, we transform the standardised data to clinical knowledge using machine learning models that could be deployable on edge devices^38^, facilitating an easier interpretation of variations and decision making of interventions for PCCTs during transport.

## Results

### Study cohort

**Fig. 1:**
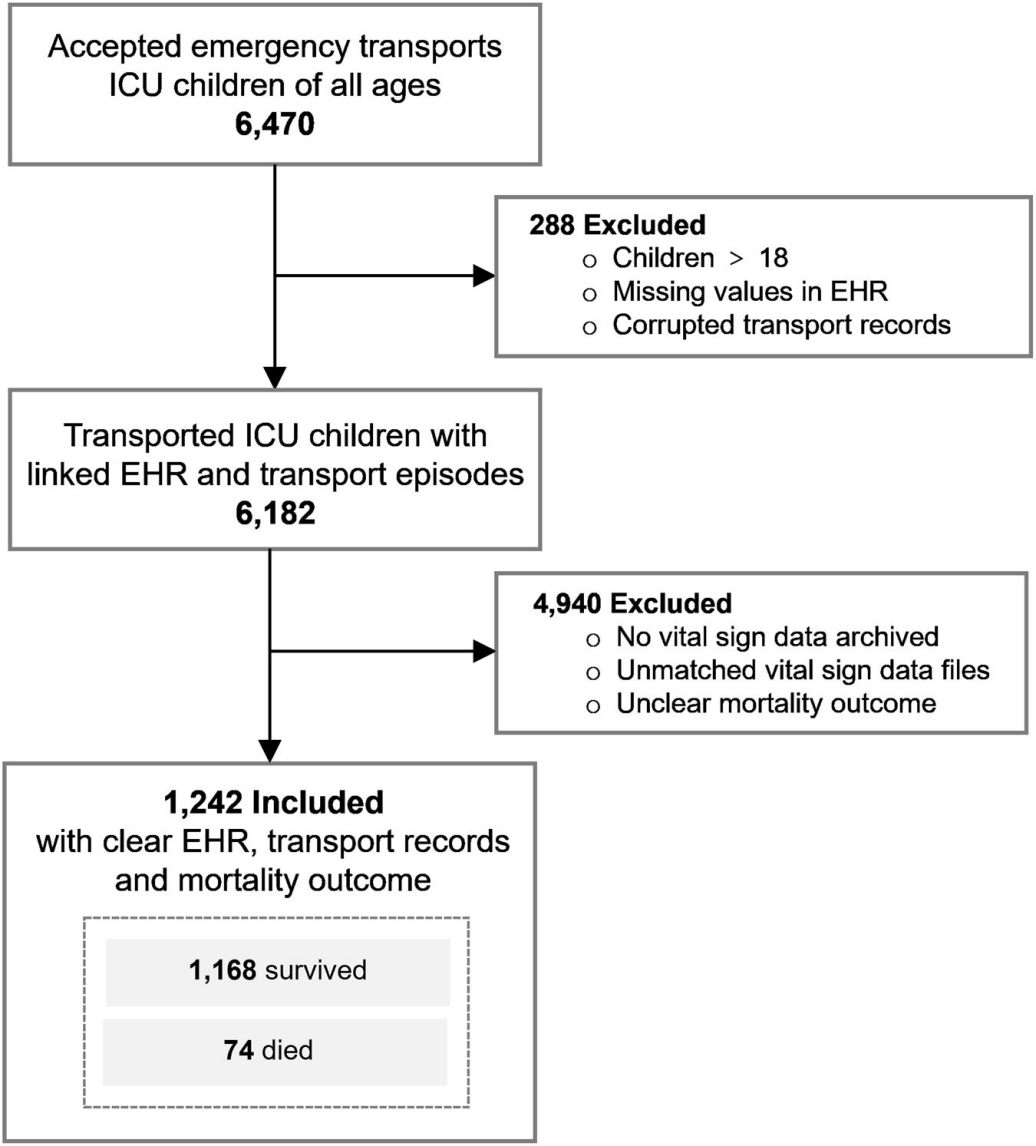
Diagram of patient screening and eligibility criteria. In total, 1242 non-repeated paediatric inter-hospital transports conducted by the CATS team between 2016 and 2021 were included.

A total of 6470 transport episodes were conducted by the Children’s Acute Transport Service (CATS), a London-based regional PCCT service, from July 2016 to May 2021^37^. Over the study period, approximately 30% of transports were eligible and included in analysis, during which vital sign data were archived throughout inter-hospital transports. The reduction in the dataset size was attributed to several factors, including technical difficulties and the unavailability of devices, leading to the exclusion of data. Figure 1 describes the flowchart of patient screening and eligibility. Patients over the age of 18, those without any archived vital sign monitoring files, and records with an excessive number of missing values in Electronic Health Records (EHR) and primary outcomes were excluded from further analysis. Finally, the study focused on 1242 non-repeated paediatric inter-hospital transports conducted by CATS between 2016 and 2021.

### Model performance

**Fig. 2:**
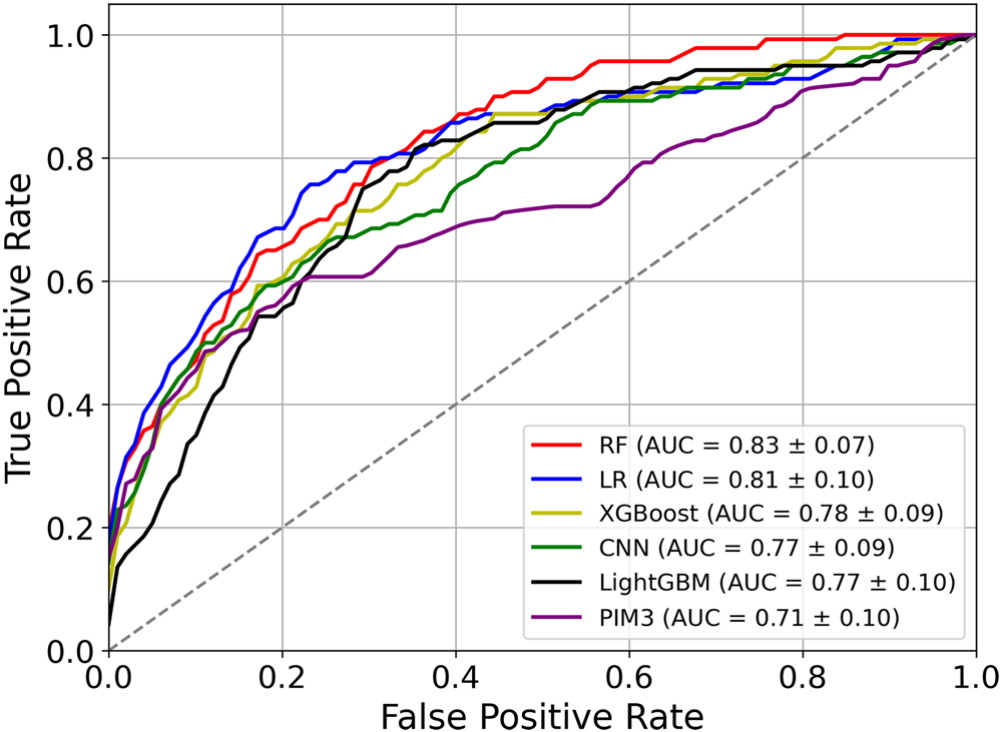
Evaluation of predictive performance using machine learning models within the PROMOT framework. Compared to PIM3, both machine learning models demonstrate improved predictive performance, as indicated by the mean AUROC and 95% confidence intervals. Random Forest (RF) and Logistic Regression (LR) exhibit the best performance among the models. AUROC Area Under the Receiver Operating Characteristic Curve.

Employing PROMPT pipeline, five ML models - Random Forest (RF), Logistic Regression (LR), Extreme Gradient Boosting Distributed Gradient-boosted Decision Tree (XGBoost), Convolutional Neural Network (CNN), and Light Gradient Boosting Machine (LightGBM) - all exhibited varied yet promising performance in predicting 30-day mortality risk within the validation cohorts (shown in Fig. 2). The area under the receiver operating characteristic curve (AUROC), Matthews correlation coefficient (MCC), average precision (AP), and other performance metrics are summarised in Table 1. The detailed meanings and interpretations of these performance metrics are explained in Supplementary Table 1 online.

**Table 1:** Comparison of model performance in the holdout test data for mortality prediction

Models^a^	AUROC^b^	MCC	PPV	NPV	Recall	F_1_-score	AP
RF	**0.83** (0.79–0.86)	0.33 (0.29–0.37)	0.59 (0.57–0.61)	0.98 (0.98–0.99)	0.79 (0.77–0.82)	**0.60** (0.56–0.62)	**0.40** (0.34–0.46)
LR	0.81 (0.76–0.85)	0.34 (0.29–0.38)	0.59 (0.58–0.60)	0.98 (0.98–0.98)	**0.80** (0.76–0.84)	**0.60** (0.57–0.63)	**0.40** (0.34–0.45)
XGboost	0.78 (0.74–0.82)	0.32 (0.27–0.38)	**0.60** (0.57–0.63)	0.98 (0.97–0.99)	0.77 (0.74–0.80)	0.59 (0.55–0.63)	0.32 (0.27–0.37)
CNN	0.77 (0.72–0.82)	0.31 (0.25–0.36)	0.58 (0.56–0.59)	0.98 (0.97–0.99)	0.78 (0.74–0.81)	0.59 (0.53–0.61)	0.25 (0.19–0.32)
LightGBM	0.77 (0.72–0.82)	0.31 (0.25–0.37)	0.59 (0.57–0.62)	0.98 (0.98–0.98)	0.76 (0.73–0.79)	**0.60**(0.55–0.63)	0.35 (0.29–0.40)
PIM3	0.71 (0.66–0.76)	0.07 (0.02–0.13)	0.46 (0.37–0.55)	0.97 (0.96–0.99)	0.56 (0.53–0.59)	0.21 (0.11–0.31)	0.33 (0.28–0.38)
RF-48h^c^	**0.81** (0.75–0.88)	**0.29** (0.23–0.35)	**0.57** (0.54–0.58)	0.99 (0.99–0.99)	**0.86** (0.80–0.91)	**0.56** (0.52–0.61)	0.15 (0.09–0.21)
PIM3-48h	0.72 (0.62–0.0.83)	0.12 (0.07–0.18)	0.52 (0.51–0.54)	0.99 (0.98–0.99)	0.65 (0.59–0.72)	0.47 (0.42–0.52)	**0.24** (0.12–0.37)

^a^*RF* Random Forest, *LR* Logistic Regression, *XGboost* Extreme Gradient Boosting Distributed Gradient-boosted Decision Tree, *CNN* Convolutional Neural Network, *LightGBM* Light Gradient Boosting Machine, *PIM3* Paediatric Index of Mortality 3.

^b^*AUROC* Area Under the Receiver Operating Characteristics Curve, *MCC* Matthew Correlation Coefficient, *PPV* Positive Predictive value, *NPV* Negative Predictive Value, *AP* Average Precision, *95% CI* 95% Confidence Interval.

^c^48h denotes mortality prediction within 48 hours of PICU admission. Unless otherwise specified, all other models represent 30-day mortality prediction performance.

Benefiting from the learning ability of PROMPT, ML models significantly increased AUROC and MCC for predicting 30-day mortality compared to the traditional method, indicating good discrimination between children who died and those who survived at 30 days. The RF model achieved the highest AUROC of 0.83 (95% Confidence Interval (CI): 0.79–0.86), while Logistic Regression (LR) also showed a commendable AUROC of 0.81 (95% CI: 0.76–0.85). This was true for 48-hour mortality prediction also (RF mode AUROC 0.81, 95% CI 0.75–0.88). In comparison, the XGBoost, CNN, and LightGBM models were slightly less accurate, with AUROCs of 0.78, 0.77, and 0.77, respectively. Furthermore, their average precision scores (≤0.35) fell short of those achieved by RF (0.40 (95% CI: 0.34–0.46)) and LR (0.40 (95% CI: 0.34–0.45)). RF and LR exhibited superior performance across different recall levels. As expected, ML models showed a significantly greater capability in predicting 48 h and 30-day mortality compared to the standard PIM3 score, which achieved a lower AUROC of 0.71 (95% CI: 0.66–0.76), F1-score of 0.21 (95% CI: 0.11–0.31), and MCC of 0.07 (95% CI: 0.02–0.13).

### Effect of features on prediction performance

**Fig. 3:**
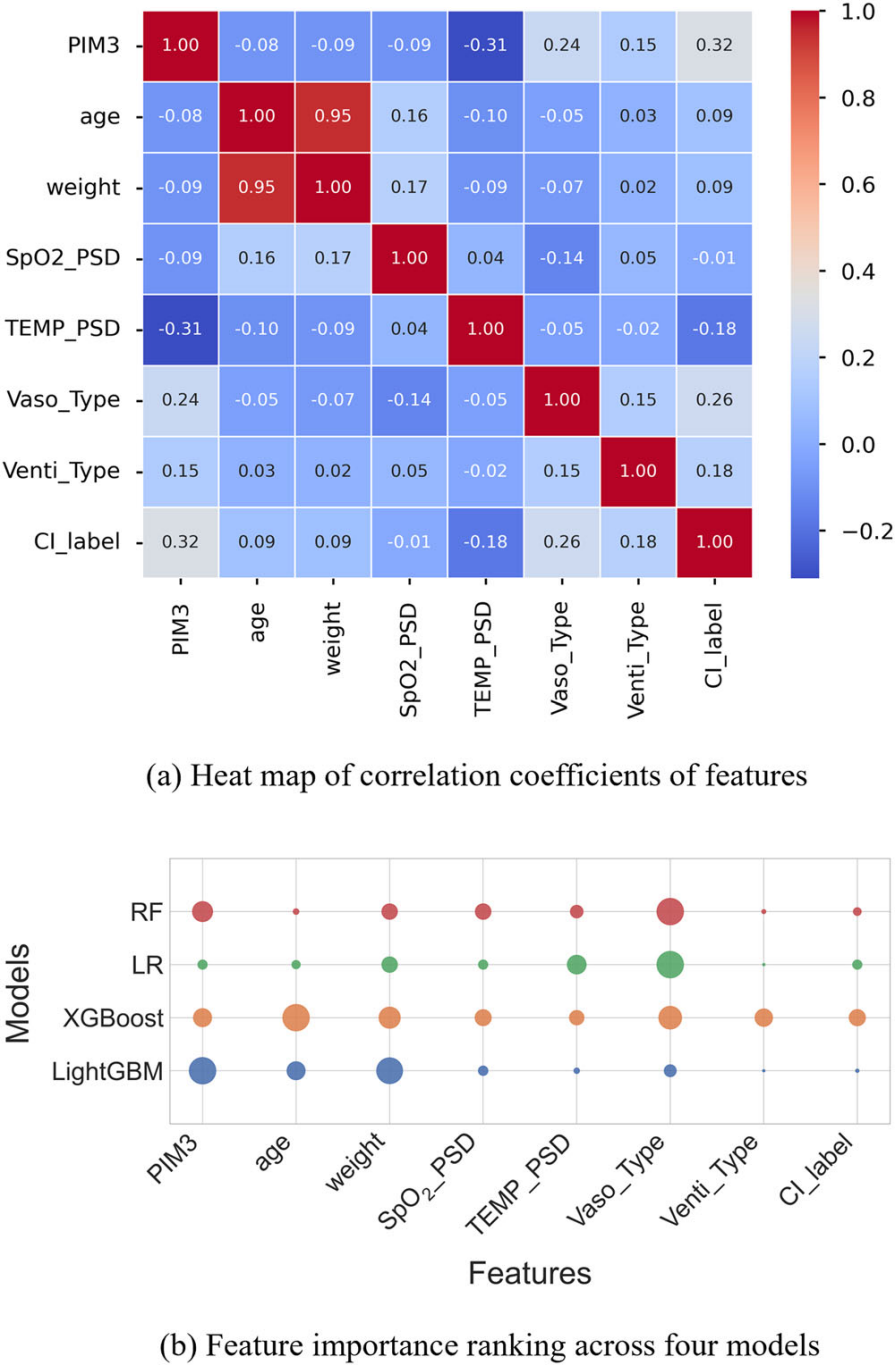
Statistical analysis of top features used in machine learning models. **a** Heatmap illustrates the Pearson’s correlation coefficients among categorical and continuous features in machine learning models. Red colours indicate stronger positive relationships, while blue colours denote stronger negative relationships. Numbers indicate positive or negative values of correlation coefficients. **b** Scaled importance rank of top eight features in four machine learning models (i.e., RF, LR, XGBoost, and LightGBM) for mortality prediction. Circle size corresponds to relative importance, while different colours represent feature importance across four models. CI_label Critical Incident label, PSD Power Spectral Density.

The correlation analysis depicted in Fig. 3a indicates significant associations among the eight important features within four ML models. These eight features were chosen for their consistent ranking as highly influential across various machine learning models. A prominent positive correlation is observed between age and weight (0.95). Additionally, the PIM3 metric is positively correlated with the Critical Incident label - CI_label - (0.32), knowing that patients with elevated PIM3 scores are likely to have a higher risk of health-related deterioration during transport. In contrast, a negative correlation of −0.31 exists between age and the Power Spectral Density (PSD) of temperature (TEMP_PSD).

Figure 3b compares the impact of selected eight features on the predictions generated by four ML algorithms. The features, including PIM3, weight, and vasoactive interventions (Vaso_type), are identified as having a significant influence on the predictive accuracy of the models. While LR tends to distribute importance more evenly among the features, ensemble techniques such as RF, XGBoost, and LightGBM demonstrate a heterogeneous importance distribution, offering insights into the varied significance of features across models. We further derived the feature importance scores within the best-performing RF model (refer to Supplementary Figs. 1 and 2 online).

### Individual-level dynamic risk assessment over transport

**Fig. 4:**
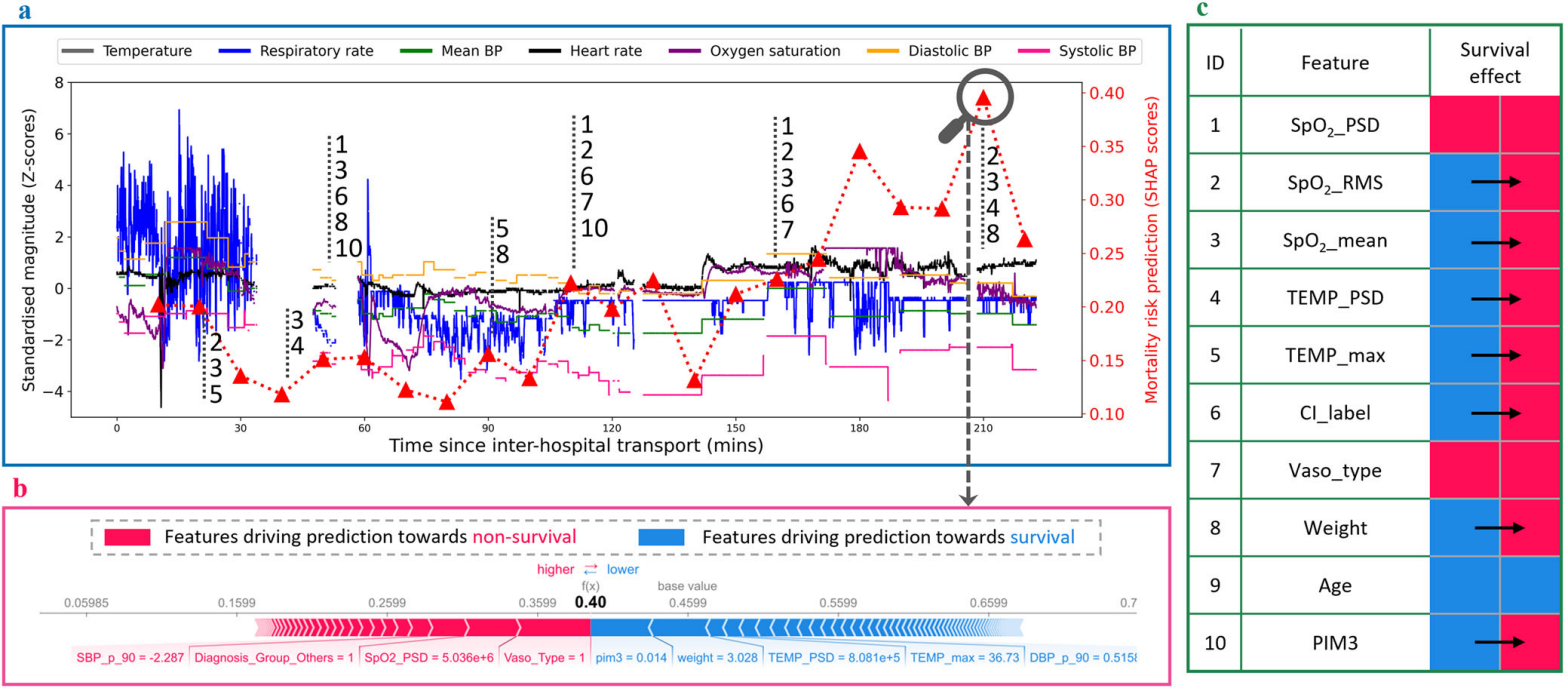
Example of co-pilot dashboard visualisation of dynamic predicted severity of illness risks and the impact of features on model prediction for a patient. **a** Plots real-time risk scores (depicted in red with triangle symbols) derived from the best-performing random forest model, employing SHapley Additive exPlanations (SHAP) analysis over the transport duration with 10-minute intervals. The left axis represents the magnitude of Z-scores of vital signs, while the right axis displays SHAP values. A higher SHAP value suggests an increased probability of the model predicting mortality. Numbers are used to identify the features; **b** explains the influence of each feature on the risk prediction for a specific time window. Features highlighted in red drive the risk prediction to the non-survival outcome; features in blue drive the prediction to the survival outcome; **c** summarised pivotal transition moments caused by a specific feature (identified by a unique ID) which drives predictions from survival to non-survival. These moments, marked by IDs in **a**, indicate time points where patient outcomes are likely to deteriorate. The transition from survival to non-survival is symbolised by a color change from blue to red. Further, we have observed certain static variables change from blue to red, with further explanation provided in the Discussion section.

The dynamic health severity risk prediction can be visually represented and explained for a specific patient at any given time. Figure 4 illustrates a representative case from the holdout test cohort. This patient was a male newborn diagnosed with metabolic acidosis. He was transferred from the general hospital to the PICU with a PIM3 score of 0.014 when the transport team arrived at the patient’s bedside. The retrieval team undertook invasive ventilation, re-intubation, repositioning of the endotracheal tube, initiated vasopressors, and prostaglandin infusion to the patient pre-transport. This patient died within 30 days after arrival at the destination PICU centre.

In this case, the patient’s mortality risk remains relatively low during the initial phase of inter-hospital transport, yet escalating as the journey nears its handover. It is observed that HR increases significantly at the 140th minute, with the patient demonstrating instability in the period from 150 to 180 min. We analysed the influence of feature variables on model predictions at the junctures of heightened risk (Fig. 4b). A combination of relatively low SBP and declining SpO_2_ drive the prediction towards non-survival, despite the administration of vasoactive support. Conversely, normal body temperature and DBP levels contribute to lowering the risk towards survival. Throughout the transport, the derived variables of SpO_2_ (ID 1,2,3 in Fig. 4c) predominantly influence the mortality prediction towards non-survival. In this case, we note that age, ventilation type, and critical incident type have relatively minor impacts on the model prediction (the patient did not experience any critical incidents during the transport), which aligns with the feature importance ranking using the RF model (shown in Fig. 3b).

### Comparing PROMPT with PIM3 in 30-day mortality prediction after inter-hospital transports to PICUs

The proposed PROMPT leverages enriched data characteristics obtained from continuous vital signs and EHR data collected during inter-hospital transports. This wealth of information enables an improved performance in tracking and predicting patients’ severity of illness during transport (Fig. 2). In contrast, current transport practices typically rely on the single time point-based PIM3 assessment, which is conducted during the early hours of arrival.

**Fig. 5:**
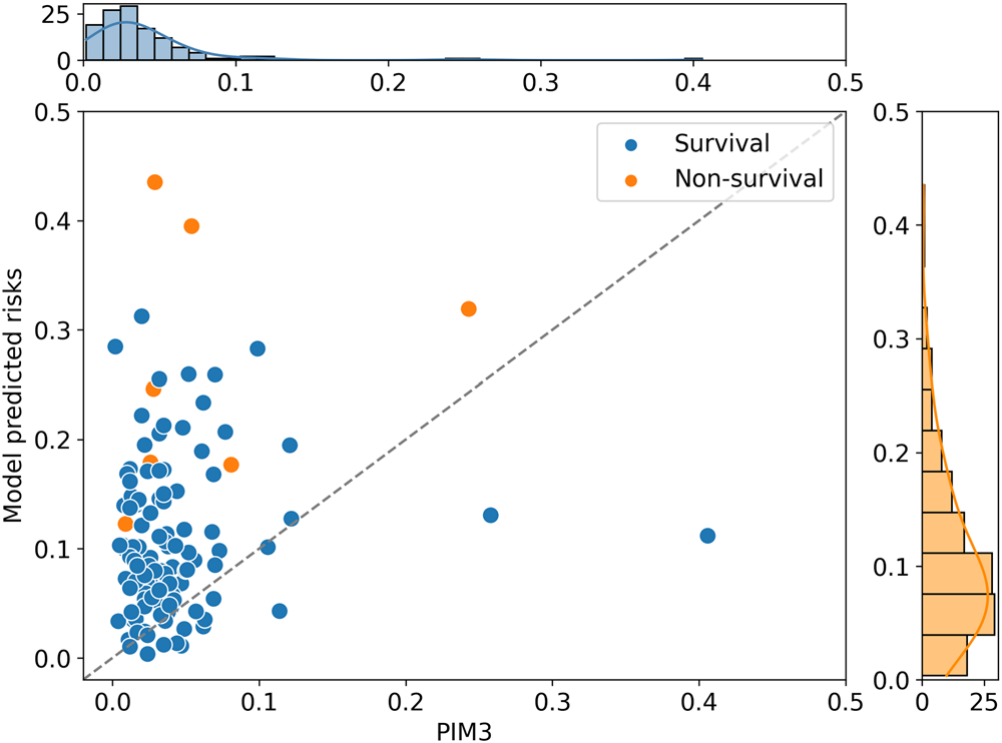
Scatter plot of patient-level mortality risk predictions using the best-performing RF and PIM3 models. Each point represents a patient, where the orange colour indicates that the particular patient died and the blue colour indicates that the patient survived. (RF random forest, PIM3 Paediatric Index of Mortality 3).

Figure 5 demonstrates the mortality prediction capabilities of our model using a holdout test cohort, comparing it against the PIM3. In this example, the best-performing RF model offers a more evenly distributed mortality risk score. It successfully identifies three patients at high risk of mortality, who, despite having low PIM3 scores (≤0.1 for two patients and ≤0.25 for one), were collected during transport. In contrast, the PIM3 scores mostly cluster below 0.15, suggesting a lower mortality risk, yet failing to account for critical incidents during transport that could significantly impact patient outcomes within 30 days post-transport, as indicated in^37^. The analysis of patients with predicted risks lower than their PIM3 scores (as shown in the Supplementary Fig. 3 online) demonstrates that the developed model exhibits improved performance in identifying low-risk cases. The clustering of points (patients) and reduced variability in predicted risks highlight its improved accuracy and consistency compared to PIM3. These findings validate the model’s superior predictive performance in scenarios involving patients with lower risks of mortality, effectively minimizing false positives.

While this comparison highlights the potential advantages of incorporating trajectory data into real-time risk assessments, we acknowledge that the differing data inputs between the two models necessitate a careful interpretation of their comparative analysis. Further, the explainability of the PROMPT facilitates ML model transparency in explaining feature influences on model output over various time windows (Fig. 4).

## Discussion

To the best of our knowledge, our study is among the first to investigate the utilisation of continuous physiological variables gathered during inter-hospital transports to the PICUs, in conjunction with EHR and specific transport episode data, to forecast 30-day mortality in critically ill children. In this work, we introduced an explainable ML pipeline named PROMPT to predict 30-day mortality risk using waveform vital signs, EHR, and transport episode data. Using PROMPT, ML models were trained on a dataset comprising 21 static EHR variables and derived features from Z-scores of vital signs for over 1200 ICU patients transported by the CATS in Central London from July 2016 to May 2021. The findings of our study demonstrated the superiority of PROMPT in predicting mortality using ML models in mortality prediction (Table 1) and model interpretability at the individual level (Fig. 4). We propose that this approach could enhance the identification of patients at risk of 30-day mortality upon arrival at the PICUs and could be continuously applied to provide real-time estimates of severity of illness during transport.

Another innovation of PROMPT lies in its ability to interpret the evolving risks of mortality with a time window for individual patients during transport (An example user interface of this “co-pilot” dashboard is shown in Fig. 4). While previous studies have concentrated on enhancing the predictive performance of ML models, they frequently overlook the importance of clinicians understanding how these models, which often function as “black boxes,” impact patient vital sign trends as well as risks over time. Using patient health and transport records, the PROMPT can dynamically assess the impact of health changes on personalised risk predictions. This study presents a real-time risk assessment score using SHAP values that updates within a 10-min (or shorter) time window, dynamically adjusting SHAP values throughout transport, as depicted in Fig. 4a. Moreover, the effects of features on patient outcomes (non-survival or survival) are elucidated in Fig. 4b, c at the individual level. This “co-pilot” dashboard dynamically tracks individual risk development trends over time, providing insights into sharp increases in risk. It has the potential to raise alerts by explaining the underlying causes at the feature level, highlighting which features are contributing to the changes and how they impact health stability. Besides, This AI solution builds on our previous study, which used Z-scores to normalize vital signs in paediatric patients, effectively minimizing age-related variations in age groups. By leveraging this approach, the basic statistics values (such as mean, max, and min) of Z-scores of vital signs informs transport clinicians about the degree of vital sign deviation from normal levels. These measures improve the transparency of the proposed AI tool by providing clinicians with explainable results and an illustrative dashboard interface. This approach demonstrates significant potential in helping transport teams quickly identify elevated risks in mobile settings.

The PROMPT facilitates the interpretation of how features influence mortality prediction using SHAP values. It was found that certain characteristics, such as SpO_2_ and vasoactive medication types, shift their impact from predicting survival to non-survival depending on the time point considered. This variability extends to other features like PIM3, weight, and the maximum temperature value (Fig. 4c, b), which can alternately contribute to predictions of both outcomes. This fluctuation underscores the complexity of interactions between features and the model over time, echoing findings from a similar study by Thorsen et al.^39^. The observed variations likely stem from the model’s ability to discern complex, non-linear relationships and patterns among the various feature variables. The model dynamically refines its predictions by adjusting the influence of specific features based on their interactions with others. This adaptability makes PROMPT more comprehensive for mortality prediction compared to snapshot-based measurements like PIM3^40^, which are often measured by the PCCTs from the time of initial patient contact within first hours at the referral hospital.

With PROMPT, our ML models, designed for inter-hospital transport of critically ill children to PICUs, address a critical gap largely overlooked in previous studies: providing real-time mortality risk prediction during the transport phase. This contrasts with the retrospective analyses often performed in other studies that rely on static data or data collected in a more controlled environment. Our models demonstrate performance metrics that are equivalent or comparable to those reported in existing studies. For instance, Kim et al.^26^, utilizing a gradient boosting decision tree model, reported an AUROC of 0.83 for predicting mortality 60 h before death with hourly vital sign measurements. Our study achieved an AUROC of 0.83 for predicting 30-day mortality post-PICU admission, emphasising the predictive power of our approach in the dynamic and often chaotic environment of inter-hospital transport. Similarly, while Feng et al.^28^ achieved an AUROC of 0.897 in early-stage mortality risk assessment among preterm infants in PICUs using deep learning models, our CNN model reported an AUROC of 0.77. Moreover, Lee et al.^11^ achieved an AUROC of 0.906 using a RF model for mortality prediction within PICUs; our study recorded an AUROC of 0.83 using the same RF model but PROMPT pipeline. The difference in performance can be attributed to the distinct contexts of each study. The dynamic nature of the transport environment, with its inherent variability in patient conditions and data quality, presents unique challenges that impact the model’s predictive accuracy. Despite the lower AUROC, our data pipeline focuses on real-time prediction and interoperability during transport. PROMPT integrates diverse data types, including continuous time-series vital signs and static clinical data, to offer dynamic and interpretable risk assessments that can inform immediate clinical interventions during transport.

Numerous studies have demonstrated the potential of ensemble learning to enhance robustness and predictive performance in critical care settings^41^. Building on the PROMPT framework, we utilized RF, XGBoost, and LightGBM as base models, leveraging their optimized hyperparameters from prior tuning, and employed LR as the meta-model to combine their predictions^42^. The results indicate that stacking achieved performance comparable to the best-performing individual machine learning models in our dataset, aligning with findings reported in a similar study^43^. Prithula also reported an AUROC of 0.72 using the CatBoost model^44^, while our proposed PROMOT pipeline with RF achieved a higher AUROC of 0.83. Similarly, their findings indicated that both the RF and CatBoost classifiers demonstrated the highest performance, whereas the stacking ensemble model showed reduced effectiveness. This trend aligns with our study, in which the RF model achieved the highest performance. However, considering the critical need for low-latency, low-complexity, and real-time explainability - essential for the transport team’s dynamic requirements and future integration into an edge-computing device on an ambulance - we prioritized the use of individual models in this study.

Patients in transports between hospitals, particularly critically ill children, present a unique challenge due to the often incomplete understanding of their underlying diagnoses and the severity of their conditions at the onset of transport^3–5^. The transport episode is characterised by the acute management of life-threatening conditions and stabilisation efforts, contrasting with the regulated environment of in-hospital care aimed at ongoing management and recovery^37^. The distinctive challenges of data variability and quality inherent in transport settings may influence the comparability of accuracy metrics.

To address the practical and technical challenges involved in transporting critically ill paediatric patients, we have created an easy-to-understand, end-to-end data pipeline powered by ML models. This pipeline incorporates conventional models, such as RF and CNN, to assess the 30-day mortality risk. Our initial investigations into Long Short-Term Memory (LSTM) models, known for their adeptness at handling sequential data^28^, revealed performance variances. These inconsistencies were linked to the high dimensionality of the feature set and prevalent missing values in the initial vital signs data, which impeded their ability to consistently surpass the performance of RF or CNN models^45^. Moreover, the urgent and time-sensitive nature of patient transport demands models that can offer real-time predictions while minimising computational complexity^46^. The necessity for reliable, real-time predictions in remote contexts - where models must often operate independently of continuous Wi-Fi or internet access - suggests a preference for light models in terms of complexity. These models stand out for their lower computational requirements, enabling timely and efficient deployment on devices capable of operating in edge-computing modes within ambulances. Considering the envisioned practical deployment scenarios, it is paramount to choose models that exhibit clear advantages in such environments^47^. Due to their relatively low computational demands and robustness, traditional models provide significant benefits over LSTM or other transformer-based models, which require more computational power and may not align well with the constraints of mobile and remote settings^48^.

Using a 10-minute interval in our dynamic risk prediction model allows us to strike a balance between providing detailed insights and maintaining fast computation when assessing the 30-day mortality risk. This approach ensures that the model remains clinically relevant without sacrificing efficiency. The purpose of this interval duration is to detect important and meaningful changes in a patient’s condition, such as fluctuations in vital signs or the effects of interventions or medications, while minimising the computational burden that comes with more frequent updates. Moreover, this time frame coincides with the standard procedure in medical practice, where a 10-minute period is deemed adequate for detecting significant changes in a patient’s condition without inundating healthcare professionals with excessive data^49–51^. Crucially, this timeframe provides adaptability, enabling modifications based on developing medical knowledge or specific situation requirements, guaranteeing that our model stays up-to-date and capable of meeting the demands of immediate clinical decision-making^52^.

Our analysis investigated the association between actual transport time (journey time on the road) and 30-day mortality outcomes. Despite the intuitive expectation that longer transport times might negatively influence patient survival, “transport time” was not among the top-ranked features in our model (see Supplementary Fig. 4 online). Specifically, it was ranked 34th in correlation with 30-day mortality predictions, suggesting a weak association between the two variables. This aligns with findings from prior studies^53,54^ that reported no significant impact of longer transport times on patient outcomes, such as 30-day mortality or hospital length of stay. Additionally, this finding is consistent with research conducted by our group, which revealed no conclusive evidence that reducing time-to-bedside significantly improves the 30-day survival rate for critically ill children^18^. While the actual transport time was analysed, incorporating the estimated transport time as a feature, particularly in the context of resource optimization or geographic challenges, could offer potential useful insights into its potential impact on patient outcomes using AI.

In our investigation, we identified several potential limitations. First, a significant challenge was the inconsistent capture of vital sign data during patient transport. Often, recording of vital signs began only upon the medical team’s arrival at the patient’s location and ceased prior to the patient’s admission to the PICU. This inconsistency, combined with the lack of standardisation in monitoring different vital signs, presented a considerable obstacle in precisely predicting mortality risks within the usual monitoring periods. Furthermore, not all transported children had their data comprehensively recorded, leading to concerns about the representativeness of our sample. Despite efforts to validate the comparability of patient characteristics within our cohort against the broader transported population, the potential for selection bias remains. Additionally, the limited generalisability due to the model being developed and validated within a single institution and over a specific period (i.e., 2016–2021) is acknowledged. While the model was effective in leveraging meaningful clinical features and high-frequency vital signs for prediction, the variation in standard-of-care practices across different institutions might require model adjustments or re-training to preserve its accuracy and applicability. For instance, our dataset documented the PIM3 score at/around the time the CATS team arrived at the patient bedside. The transport phase included logging of critical incidents. The challenge of integrating and analysing data from multiple sources for model validation underscores the significant infrastructural and logistical challenges in extending the model’s application to a wider clinical context. This limitation underlines the necessity for future research to focus on improving the model’s adaptability and validating its performance in varied healthcare settings to ensure its generalisability and efficacy in clinical decision-making.

Another limitation pertains to the interpretability and causal implications of model predictions. While the study endeavoured to enhance model interpretability, it is vital to recognise that the associations identified between specific features and health severity risks, such as 30-day mortality post-arrival at destination PICUs, are correlative rather than causative. This distinction implies that modifying a feature significantly influencing the model’s prediction does not guarantee an altered patient risk or outcome. For example, the identification of certain clinical interventions or critical incidents in a patient’s history might correlate with increased risk scores, yet altering these based on model suggestions alone, without considering the clinical context, could prove imprudent. This emphasises the importance of incorporating clinical judgement and expertise into model refinement, aiming for further research to investigate how these predictive models can support clinical decision-making effectively, without solely dictating patient care strategies, particularly in the exigent and variable transport environments. Although obtaining new data for validation is challenging, we are actively working to address these limitations. This includes collaborating with other institutions to replicate our data extraction methods and using more recent data from our own institution for further validation. These efforts aim to improve the model’s generalisability and practical value.

One potential limitation is the generalizability of the proposed method across different ethnicities, as the study population is primarily drawn from south-east England. This may affect the model’s applicability to populations with diverse demographic and clinical characteristics in other regions or countries. Limited access to geographically diverse datasets remains a challenge. To address this, efforts are underway to expand the dataset by incorporating cohorts from south-east England and other parts in the UK, while also exploring collaborations with external institutions to enhance data diversity.

Future work will focus on seamlessly integrating this tool into edge-computing devices on ambulances, leveraging its low computational complexity and near real-time response capabilities, prior to rigorous clinical validation. By providing dynamic risk scores and pinpointing specific vital signs driving changes in risk, the tool enables transport teams to rapidly identify and address high-risk situations, ensuring reliable, real-time functionality even in resource-limited environments. Furthermore, once connected to secure cloud services, the tool can support seamless handovers by delivering comprehensive, time-stamped patient data and risk trends directly to destination hospitals. This ensures that receiving clinicians have immediate access to actionable information, enabling accurate prioritization of interventions, streamlined triaging, and improved outcomes for critically ill children. Extensive validation studies are planned to rigorously evaluate the tool’s performance in real-world clinical settings, ensuring its reliability and effectiveness in enhancing paediatric care.

## Methods

### Study population and data sources

**Fig. 6:**
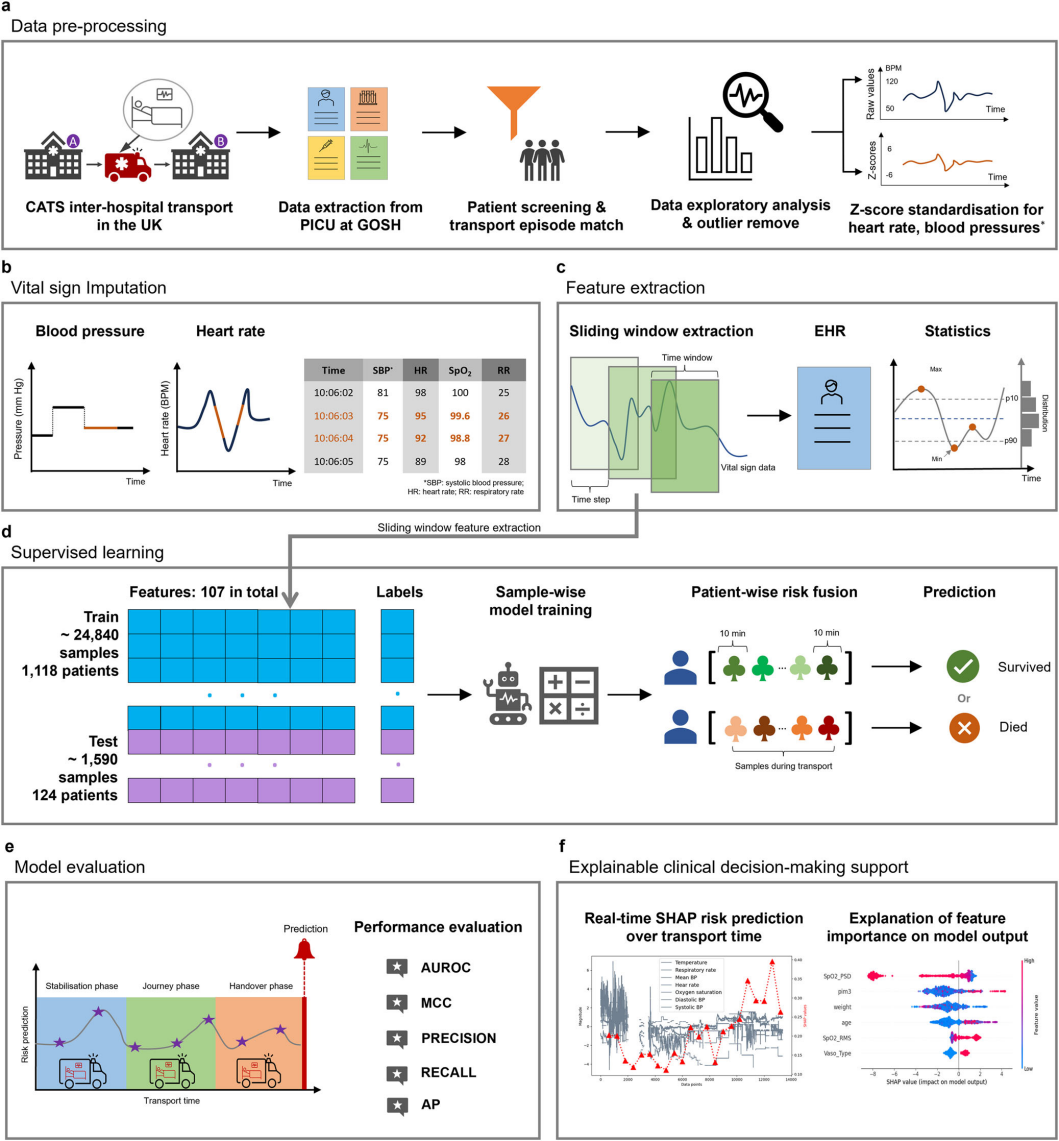
Overview and methodology of PROMPT: from raw data to explainable clinical decision-making support. **a** Raw data extracted from PICU were filtered using the inclusion and exclusion criteria. Clinically implausible values were removed, and raw data were pre-processed following data exploratory analysis. Cleaned data were converted to standardised data using the Z-score approach. **b** Imputation was applied to fill in missing values in vital signs time-series data using variable-specific methods for every time point. **c** Sliding window extraction scheme was performed to extract balanced samples to mitigate the problem of mining imbalanced datasets (samples extracted from deceased patients are minority class). Static features (i.e., EHR and transport episode data) and statistical features extracted from high-frequency data (i.e., physiological time-series data) were integrated into feature vectors. Each feature vector represents one patient’s characteristics for a specific period. **d** Balanced samples were employed to train the binary machine learning models, and the predicted mortality risk probabilities were fused to generate the 30-day mortality risk score in validated patients. **e** The modelling performance was evaluated using widely used metrics for evaluation of machine learning models on imbalanced datasets such as Area Under the Receiver Operating Characteristic Curve (AUROC), Recall, Matthews Correlation Coefficient (MCC) and recall, etc. **f** SHapley Additive exPlanations (SHAP) algorithm was employed to explain features that drive individual-specific predictions and visualise SHAP risk scores in real-time during transport.

This retrospective cohort includes 1242 patients who were transported from general hospitals to PICUs in central London, with at least 10 min of archived vital signs data. The medical and vital sign information of all patients collected between July 2016 to May 2021 were used for model development. Table 2 presents the characteristics of overall transported patients and study cohort. The study cohort comprised children with a median age of 8 months (Interquartile Range, IQR: 0–53 months), predominantly diagnosed with respiratory (34.8%) and cardiovascular conditions (25.7%). The median PIM3 predicted mortality risk was 3.3% (IQR: 2–4.9). Critical incidents during transport, either due to patient deterioration or equipment malfunction, were reported in 14.9% of cases. Out of the 1242 patients, 1168 survived and 74 died within 30 days of PICU admission (20 died within 48 h of PICU admission). The median transport time was 206 min (IQR: 160–258 min). A median of 1.12 (IQR: 0.69–1.73) monitoring hours per patient was collected from the study population, totalling 1722.53 h of vital signs monitoring data. This cohort’s EHR and vital signs data were utilised in the development of ML models (Fig. 6 describes study overview and methodology).

**Table 2:** Characteristics of demographics, clinical results, and transport episodes of overall transported patients and study population

Characteristics^a^	All transported patients (*n* = 6182)	Patients analysed in this study (*n* = 1242)
Age, months/years		
≤1 m (newborn)	2145 (34.7%)	471 (37.9%)
1–≤12 m (infant)	1270 (20.5%)	254 (20.5%)
1–≤4 y (pre-school child)	1148 (18.6%)	218 (17.6%)
4–≤11 y (school child)	976 (15.8%)	180 (14.5%)
11–≤18 y (adolescent)	643 (10.4%)	119 (9.5%)
Gender		
Male	3449 (55.8%)	574 (46.2%)
Diagnosis group		
Respiratory	2220 (35.9%)	432 (34.8%)
Cardiovascular	1409 (22.8%)	319 (25.7%)
Neurological	1069 (17.3%)	229 (18.4%)
Infection	625 (10.1%)	80 (6.4%)
Gastrointestinal	461 (7.5%)	55 (4.4%)
Trauma	321 (5.2%)	16 (1.3%)
Others	76 (1.2%)	111 (9.0%)
PIM3 predicted mortality risk^b^		
≤1%	392 (6.3%)	75 (6.0%)
1%–≤3%	2282 (36.9%)	422 (33.9%)
3%–≤5%	2022 (32.7%)	433 (34.9%)
5%–≤10%	1016 (16.4%)	219 (17.6%)
10%–≤15%	194 (3.0%)	39 (3.2%)
15%–≤30%	171 (2.8%)	31 (2.5%)
>30%	105 (1.7%)	23 (1.9%)
Invasive ventilation		
Yes	4208 (68.1%)	889 (72.4%)
Vasoactive agent infusion		
Yes	1792 (28.9%)	390 (31.4%)
Inhaled nitric oxide		
Yes	198 (3.2%)	31 (2.5%)
Critical incident^c^		
Patient or Equipment related	824 (13.3%)	186 (14.9%)
Overall transport time, minutes^d^		
≤180	2203 (35.6%)	403 (32.5%)
180–360	3744 (60.6%)	804 (64.7%)
>360	235 (3.8%)	35 (2.8%)

^a^Data are *n* (%). Patients with multiple transports are excluded.

^b^The PIM3 score was measured at first face-to-face contact with the transport team, usually within the first hour of the stabilisation phase at the patient’s bedside.

^c^Patient or equipment related critical incidents occurred during transports are associated with adverse events in transported children.

^d^Overall transport time consists of stabilisation time, patient journey time and handover time.

### Data collection and processing

The collected data comprised categorical and numerical datasets (Table 3). Patient demographic information, including age, gender, diagnosis group, along with clinical data such as the use of invasive mechanical ventilation and vasoactive drugs were gathered during inter-hospital transports. PIM3 score was calculated using variables collected at/around the time the CATS team arrived at the patient bedside. Numerical data included physiological variables: systolic blood pressure (SBP), diastolic blood pressure (DBP), mean blood pressure (MBP), heart rate (HR), respiratory rate (RR), peripheral capillary oxygen saturation (SpO_2_), and body temperature, which were continuously monitored and automatically recorded on the EHR throughout the transport. The vital signs of critically ill patients, characterised by their high frequency, were extracted and processed by a data engineering team within the CATS transport team and at the transport destination hospitals.

**Table 3:** List of collected EHR and vital signs data in the study cohort

Category	EHR and vital signs data	Data type	Range	Missing (%)^a^
Patient demographics	Age, weight, gender	Categorical	0,1	
Medical diagnosis	Primary disease diagnosis, paediatric index of mortality 3, critical incidents^b^	Categorical	0,1	
Interventions^c^	ECMO, mechanical ventilation, vasoactive medicines, Inhale nitric oxide intervention	Categorical	0,1	
Vital signs	Heart rate (bpm),	Numerical	[0,300]	2.85
	Systolic BP (mmHg),	Numerical	[0,270]	9.25
	Diastolic BP (mmHg),	Numerical	[0,250]	9.30
	Mean BP (mmHg),	Numerical	[0,250]	8.76
	Respiratory rate (breaths/min),	Numerical	[0,180]	3.11
	SpO_2_ (%)	Numerical	[0,100]	2.87
	Body temperature (°C)	Numerical	[20,45]	12.5

^a^Missing rates in the collected data were calculated after data extraction and initial data cleaning processes generated from the data registry.

^b^Critical incident during paediatric transport refers to an unexpected event or situation that arises while transporting a paediatric patient, which poses a significant risk to the patient’s health or well-being due to patient-related deterioration or medical equipment failures.

^c^Intervention records represent care support administered by the paediatric critical care transport teams.

EHR and vital signs are frequently collected in mobile settings, leading to the presence of abnormal records and missing values, often resulting from medical staff errors or unexpected observation disruptions due to acute treatment. To address these issues, we utilised imputation and filtering methods for outliers, missing values, and diverse feature ranges (Fig. 6b). Our method also included data cleaning to standardise the variability in each physiological signal and remove outliers or invalid data. This process was critical because the initial recordings of vital signs fell within specific ranges rather than discrete values (refer to Supplementary Table 2 online).

In the data pre-processing stage, we standardised vital signs using the Z-score approach for all patients. This normalisation is particularly important in paediatric populations, where vital signs such as blood pressure and heart rate exhibit significant variability and age-dependent ranges. By normalising the vital signs, we mitigate age-related variability in vital signs, leading to more reliable model training and evaluation based on relative deviations rather than widely varying age-specific vital sign data. A comprehensive description of our Z-score standardisation method is detailed in our preliminary retrospective study^37^.

The study adhered to the protocols of the Data Research, Innovation, and Virtual Environments (DRIVE) unit at Great Ormond Street Hospital (GOSH), ensuring compliance with ethical guidelines for processing patient data. All data handling was conducted within the secure Digital Research Environment^55^ and underwent both de-identification and anonymization processes. Additionally, access to the de-identified patient and transport records was strictly controlled.

### Feature generation

In the PICU setting, mortality events are infrequent relative to survival cases. Our study revealed a 30-day PICU mortality rate of approximately 6% (1.6% mortality within 48 h), indicating a significant class imbalance. To address this, we employed a sliding time window technique (with a duration of 10 min and a step size of 50 data points) to augment the minority class by extracting samples from deceased cohort. For most classes, we just use non-overlapping sliding time window to generate samples from survival patients. In this case, the number of samples with deceased patients are approximately equal to that of samples with surviving patients (Fig. 6c).

Subsequent to the application of the sliding window technique, features were generated from the samples (10 min of Z-scores) as inputs. EHR data were used as static features, including demographics, medical support information, and clinical scores. For the continuous Z-scores of vital signs, thirteen types of features were generated for each time window, encompassing statistical measures such as mean, standard deviation, and entropy measures^56^. These features, derived from both EHR and Z-scores of vital signs, were assembled into a comprehensive feature set (refer to Supplementary Table 3 online).

### Development of the model

The model’s development incorporated five foundational individual ML models (i.e., RF, LR, XGBoost, CNN and LightGBM). The principal focus was on predicting mortality within 30 days post-admission to the PICU after inter-hospital transport. The patients were divided using the holdout method, allocating 90% to the training dataset and 10% to the holdout dataset through random sampling. Meanwhile, the approximate death rate of 6% observed in the original dataset was maintained in both the training and holdout datasets. In preparing samples for training and testing, positive samples were derived from all deceased patients using a sliding time window approach, ensuring an approximate equal number of positive and negative samples to mitigate the imbalanced learning. Negative samples were extracted from all surviving patients (without the sliding method). We rigorously ensured that no sample data from the same patient was shared between the training and test sets, thereby preventing data leakage and maintaining the integrity of the validation process.

Throughout the training phase, a sample-wise approach was applied to train the classifiers, enabling the models to learn patterns from patient samples. Samples extracted from patients allocated to the training dataset were used for model training, and a five-fold cross-validation was employed to fine-tune the model hyperparameters. During the cross-validation process, a random search approach was applied to systematically explore combinations of key hyperparameters in different machine learning models. The optimal parameters were selected based on the highest average performance across the folds, using metrics such as AUROC to balance sensitivity and specificity. To predict patient-level mortality risk, the mortality risk probabilities of all available samples predicted from ML models from the same patient were averaged (Fig. 6d).

### Model evaluation

Model performance was assessed using several metrics, including the AUROC, MCC, AP, Positive Predictive Value (PPV), and Negative Predictive Value (NPV) (Fig. 6e). These metrics provided a comprehensive evaluation of the models’ ability to differentiate between survivors and non-survivors in an unbalanced dataset. The performance of the classifiers was compared against that of the PIM3.

### Model prediction explanation

We employed a SHAP algorithm to explain our model prediction^57^. SHAP values partition the prediction result of every sample into the contribution of each constituent feature value - it explains the contribution of each feature value that drive model prediction^39^. This approach not only reveals the impact of feature values on the model’s predictions but also facilitates an understanding of how changes in these values influence clinical outcomes (Supplementary Fig. 5 online). By focusing on the RF model, we demonstrated the application of SHAP values in visualising patient-specific continuous risk predictions and the contributory role of individual features within the context of their interactions, thereby offering insights into real-time health risk trends over transport time (Fig. 6f). An explanation of SHAP value changes and their influence on model predictions is provided in Supplementary Table 4 online.

### Ethics consideration

Formal ethical approval was waived since data were collected as part of routine care and anonymized before analysis, which was covered by generic research database approval (17/LO/0008) from the London - South East Research Ethics Committee.

## Supplementary information

Supplementary Information

## Data Availability

The data that support the findings of this study are available from Children’s Acute Transport Service and Great Ormond Street Hospital in London. Restrictions apply to the availability of these data, which were used under license for this study. Data are available from the corresponding author with the permission of Great Ormond Street Hospital.

## Code availability

Analysis code used in this project is available at Zenodo: https://zenodo.org/records/11283103. This study uses Python (v3.10.9) for the analysis, and the versions of the libraries (e.g., SHAP, NumPy, Pandas, scikit-learn, and Seaborn) are detailed in Supplementary Table 5 online.
