# Supplementary material for: Dynamic mortality prediction in critically Ill children during interhospital transports to PICUs using explainable AI
Source: NPJ Digit Med. 2025 Feb 17;8:108. doi: 10.1038/s41746-025-01465-w (PMC11832768; doi:10.1038/s41746-025-01465-w)
Supplement: Supplementary file 1 — Supplementary Information [file 41746_2025_1465_MOESM1_ESM.pdf]

Supplementary Information

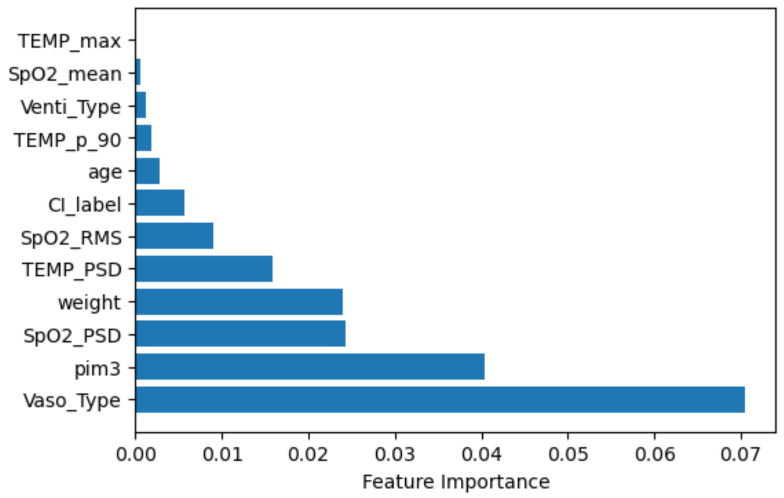

Supplementary Figure 1: Ranked top 12 features using the random forest model. Feature importance is calculated based on the mean decrease in impurity (MDI) from each feature. Essentially, it measures how much each feature decreases the uncertainty of the model (e.g., Gini impurity for classification tasks). It is computed during the construction of the random forest by adding up the weighted impurity decrease for all nodes a feature is used to split.

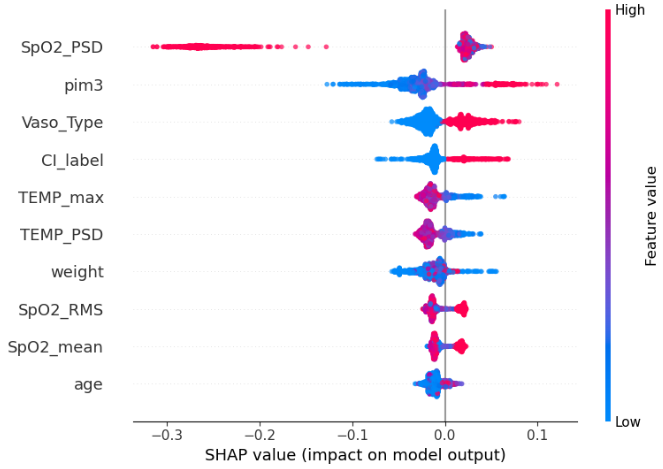

Supplementary Figure 2: Contribution of features to model predictions using the SHapley Additive exPlanations (SHAP) analysis. The colours represent feature values: red for greater values and blue for smaller ones. A positive SHAP value for a feature indicates that higher values of that feature contribute to driving the model prediction towards the outcome of death, while a negative SHAP value for a feature indicates that lower values of that feature contribute to predicting the survival class.

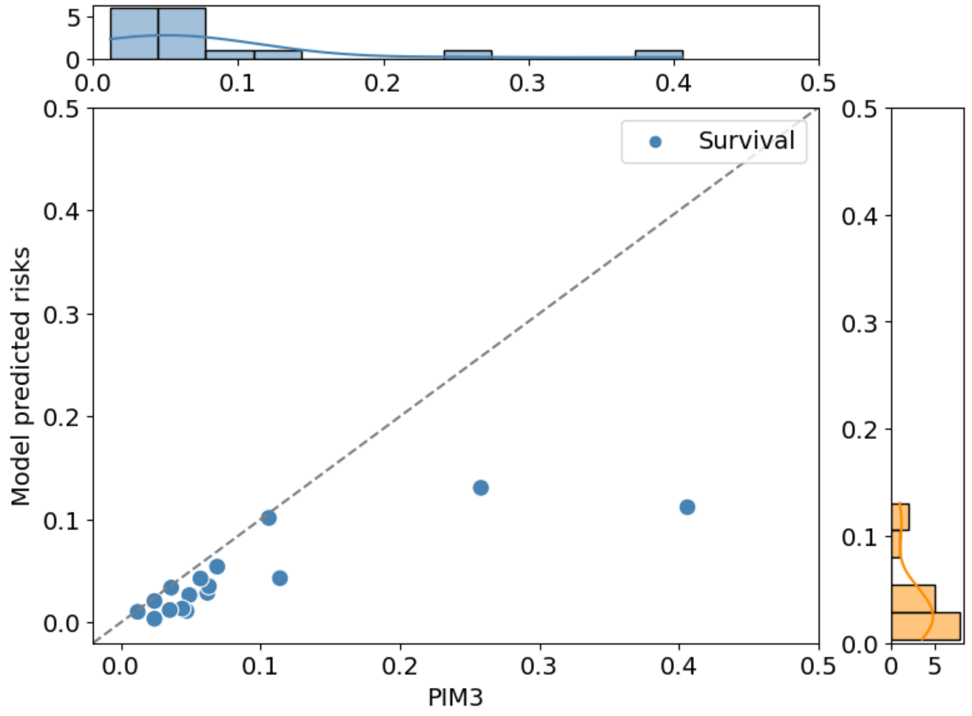

Supplementary Figure 3: Scatter plot of identified patients whose AI predicted risks were lower than their PIM3 scores (please note that no non-survival patients were identified in this case). Each point represents a transported patient, all of whom survived within 30 days after admission to the PICU centres. The clustering of points near the diagonal line suggests the developed model provides more consistent predictions with reduced variability for low-risk patients, compared to the broader variability typically associated with PIM3.

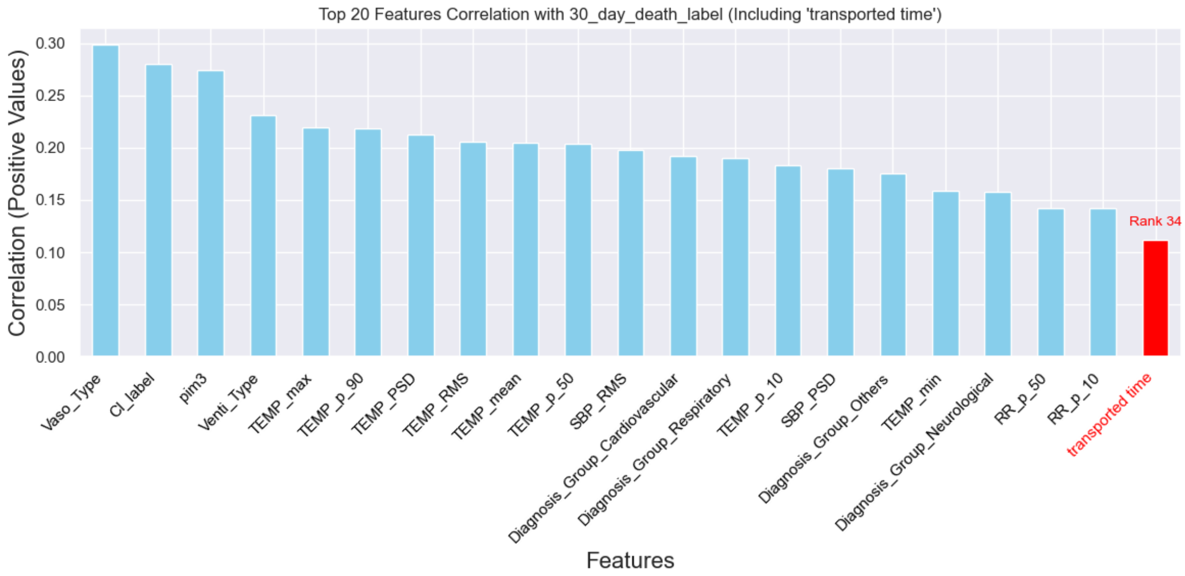

Supplementary Figure 4: Top 20 features ranked by their correlation with the 30-day mortality outcome, including "transport time". The feature "transport time" is ranked 34th, indicating a low correlation with the outcome and suggesting limited predictive significance in this study.

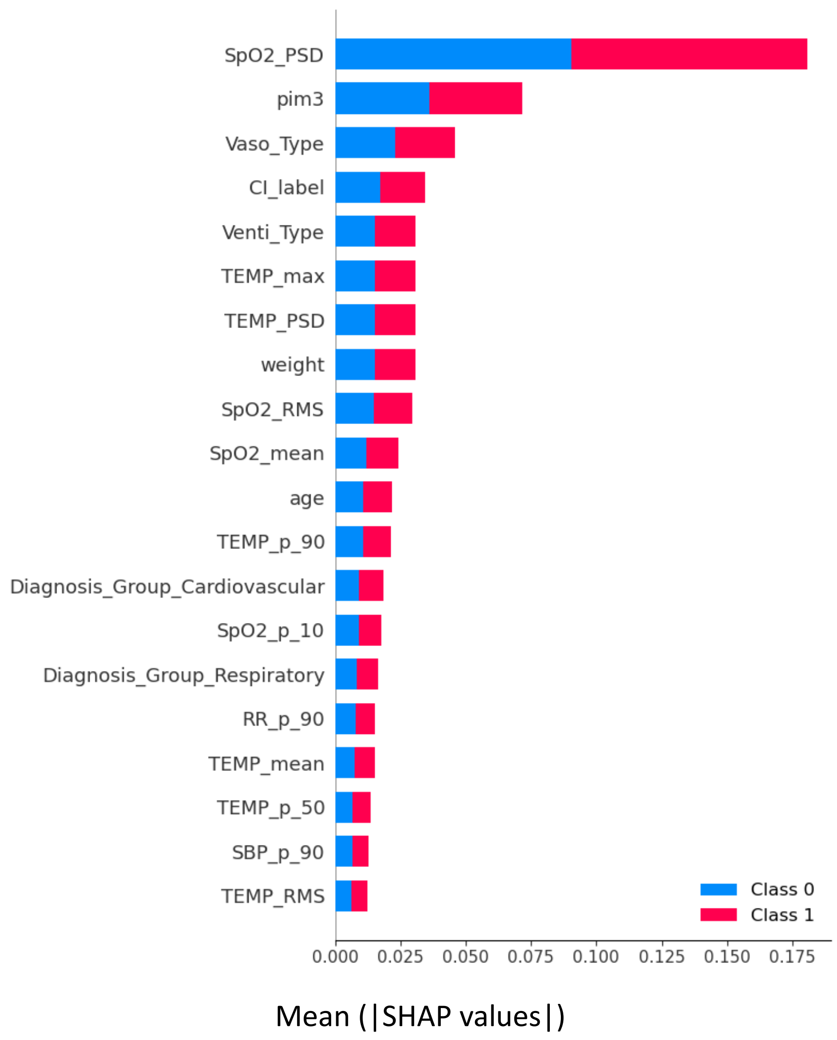

Supplementary Figure 5: The SHAP values, based on game theory, aim to explain the prediction of an instance by computing the contribution of each feature to the prediction. SHAP values provide both local explanations (for individual predictions) and global explanations (across the entire dataset). It does this by comparing what a model predicts with and without each feature, but unlike MDI, it does so by taking all possible combinations of features into account.

Supplementary Table 1. Explanation of model performance metrics used in this study.

Metric	Concept	Meaning	Interpretation
AUROC	Area Under the Receiver Operating Characteristic Curve	Measures the ability of the model to distinguish between classes. A higher value indicates better discrimination between positive and negative outcomes.	Higher AUROC (closer to 1) indicates the model can effectively differentiate between patients at high and low mortality risk.
MCC	Matthews Correlation Coefficient	Represents the correlation between predicted and actual classifications. Values range from -1 (inverse prediction) to +1 (perfect prediction), with 0 indicating no better than random chance.	A higher MCC indicates strong agreement between predicted and true classes; low or negative MCC indicates poor predictive reliability.
PPV	Positive Predictive Value	Indicates the probability that positive predictions are actually correct.	Higher PPV (closer to 1) indicates that most of the predicted high-risk cases are actual high-risk patients.
NPV	Negative Predictive Value	Indicates the probability that negative predictions are actually correct.	Higher NPV (closer to 1) suggests that most of the predicted low-risk cases are indeed low-risk.
Recall	Recall (Sensitivity)	Represents the proportion of actual positives that are correctly identified by the model.	Higher Recall (closer to 1) means the model can identify the majority of true high-risk cases, reducing false negatives.
F1-score	F1-score	The harmonic mean of Precision and Recall, balancing the two in cases where both false positives and false negatives are considered.	A high F1-score indicates balanced and reliable performance in identifying high-risk cases, taking both false positives and negatives into account.
AP	Average Precision	Summarizes the precision-recall curve, where a higher value indicates better overall model performance in terms of precision across recall levels.	A high AP score indicates good precision at various recall thresholds, reflecting consistency in correct positive predictions across recall levels.

Supplementary Table 2. Data cleaning and preparation.

Data Type	Exclusion Criteria	Correction Method
Heart Rate	Less than 30 or greater than 300 beats per minute	Interpolated using linear regression
Body Temperature	Below 25°C or above 41°C	Abnormal readings adjusted to 36.5°C; missing values imputed with 36.5 °C
Oxygen Saturation (SpO_2)	Below 30%	Interpolated using linear regression
Blood Pressure	No specific range criteria	Backward interpolation to impute missing values

Supplementary Table 3. Description of model input features.

Category	Feature	Feature description
Categorical data	age	Age at admission
	weight	Weight
	pim3	Paediatric Index of Mortality 3
	gender	Gender
	NO_Type	Inhale nitric oxide support (Yes/No)
	ECMO	ECMO support (Yes/No)
	Venti_Type	Invasive mechanical ventilation or Non-invasive ventilation support
	Vaso_Type	Vasoactive support (Yes/No)
	Diagnosis_Group_Cardiovascular	Yes/No
	Diagnosis_Group_Gastrointestinal	Yes/No
	Diagnosis_Group_Infection	Yes/No
	Diagnosis_Group_Neurological	Yes/No
	Diagnosis_Group_Respiratory	Yes/No
	Diagnosis_Group_Others	Yes/No
	CI_label	Critical Incident Event (Yes/No)
Numeric data (vital signs: systolic blood pressure (SBP), diastolic blood pressure (DBP), mean blood pressure (MBP), heart rate (HR), respiratory rate (RR), peripheral capillary oxygen saturation (SpO_2), body temperature)	mean	Mean value
	std	Standard deviation
	max	Maximum of signal x
	min	Minimum of signal x
	p_10	10th percentile value
	p_90	90th percentile value
	p_50	50th percentile value (= median)
	kurtosis	Kurtosis
	skewness	Skewness
	RMS	Root mean square (RMS)
	Peak	Peak value: $0.5 * (\max(x_i) - \min(x_i))$
	PSD	Power Spectral Density (PSD): Summation of power of signal $x_{(i)}$
	Fuzzy entropy	Uncertainty or randomness in time series

Note: For categorical data, they are directly used as features in the feature vector. For numeric data - Z-scores of high-frequency vital signs, features are calculated for each kind of vital signs (i.e., SBP, DBP, MBP, HR, RR, SpO_2, and body temperature) respectively.

Supplementary Table 4. Explanation of SHAP Value Types and Their Influence on Model Predictions Over Time.

SHAP Value Type	Meaning	Impact on Prediction	Interpretation Over Time
Positive SHAP value	Indicates the feature contributes positively, pushing the prediction towards a specific outcome (e.g., survival).	Drives the model's prediction towards a positive outcome.	If maintained or increasing over time, the feature continually supports the positive prediction.
Increased Positive SHAP value	The positive SHAP value has increased, meaning the feature's positive influence on the outcome is getting stronger.	Strengthens the model's confidence in a positive outcome.	An increasing trend over time shows the feature's positive impact is becoming more significant for the outcome.
Decreased Positive SHAP value	The positive SHAP value has decreased, meaning the feature's positive influence on the outcome is lessening.	Weakens the model's confidence in a positive outcome.	A decreasing trend over time indicates a diminishing positive impact on the outcome.
Negative SHAP value	Indicates the feature contributes negatively, pushing the prediction towards an alternative outcome (e.g., non-survival).	Drives the model's prediction towards a negative outcome.	If maintained or increasing over time, the feature continually supports the negative prediction.
Increased Negative SHAP value	The negative SHAP value has increased (moved further negative), meaning the feature's negative influence on the outcome is getting stronger.	Strengthens the model's confidence in a negative outcome.	An increasing negative trend shows the feature's influence towards the negative outcome is intensifying.
Decreased Negative SHAP value	The negative SHAP value has decreased (moved closer to zero), meaning the feature's negative influence on the outcome is weakening.	Weakens the model's confidence in a negative outcome.	A decreasing trend suggests the feature's negative impact on the outcome is weakening over time.

Supplementary Table 5. Free and open-source software used in analysis and modelling.

Software Package	Version	License Type	Description	Citation
Python	3.10.9	PSF License	Programming language used for data analysis	Python Software Foundation. Python Language Reference, version 3.10.9. Available at http://www.python.org.
NumPy	1.23.5	BSD License	Numerical computing library	Harris, C.R., Millman, K.J., van der Walt, S.J., et al. (2020). Array programming with NumPy. Nature, 585, 357–362.
pandas	1.5.3	BSD License	Data manipulation and analysis library	McKinney, W. (2010). Data Structures for Statistical Computing in Python. Proceedings of the 9th Python in Science Conference, 51-56.
scikit-learn	1.2.1	BSD License	Machine learning library	Pedregosa, F., Varoquaux, G., Gramfort, A., et al. (2011). Scikit-learn: Machine Learning in Python. Journal of Machine Learning Research, 12, 2825-2830.
TensorFlow	1.7.5	Apache-2.0	Deep learning framework	Abadi, M., Barham, P., Chen, J., et al. (2016). TensorFlow: A System for Large-Scale Machine Learning. OSDI'16: Proceedings of the 12th USENIX Conference on Operating Systems Design and Implementation, 265-283.
Keras	2.12.0	MIT License	Deep learning library	Chollet, F., et al. (2015). Keras: The Python Deep Learning Library. https://keras.io.
Matplotlib	3.7.0	PSF License	Plotting library for Python	Hunter, J.D. (2007). Matplotlib: A 2D Graphics Environment. Computing in Science & Engineering, 9(3), 90-95.
Seaborn	0.12.2	BSD License	Statistical data visualization library	Waskom, M., et al. (2020). Seaborn: Statistical Data Visualization. Journal of Open Source Software, 6(60), 3021.
SHAP	0.41.0	MIT License	Explainable AI visualization library	Lundberg, S.M., & Lee, S.-I. (2017). A Unified Approach to Interpreting Model Predictions. Advances in Neural Information Processing Systems, 30, 4765-4774.
